# Omeprazole-induced and pantoprazole-induced asymptomatic hyponatremia: a case report

**DOI:** 10.1186/s13256-020-02423-8

**Published:** 2020-06-29

**Authors:** Isabel J. B. van der Zalm, Tom J. M. Tobé, Susan J. J. Logtenberg

**Affiliations:** Department of Internal Medicine, Diakonessenhuis, Bosboomstraat 1., 3582 K.E. Utrecht, The Netherlands

**Keywords:** Hyponatremia, Omeprazole, Pantoprazole, SIADH, Re-challenge

## Abstract

**Background:**

Hyponatremia is the most common electrolyte disorder. Thiazides, antidepressants, antipsychotic drugs, and antiepileptic drugs are well-known causes of hyponatremia. Proton pump inhibitor use is a rare cause of hyponatremia and, when reported, it is due to one specific proton pump inhibitor, mostly omeprazole.

**Case presentation:**

A 67-year-old Caucasian male was referred to our out-patient clinic because of hyponatremia (127 mmol/L) found at routine laboratory examination. He had consulted his general practitioner because of abdominal pains. No other symptoms were present. At physical examination, he appeared euvolemic and had no abdominal tenderness. Besides omeprazole for reflux esophagitis he used no medication. Additional laboratory results included: serum osmolarity 274 mOsmol/kg, urinary osmolarity 570 mOsmol/kg, and urinary sodium 35 mmol/L. Other causes of hyponatremia were excluded and we diagnosed hyponatremia due to the syndrome of inappropriate antidiuretic hormone secretion secondary to use of omeprazole. Omeprazole was replaced by ranitidine after which his serum sodium levels normalized to 135 mmol/L. During follow-up, because of persistent reflux complaints despite ranitidine use, ranitidine was switched to another proton pump inhibitor: pantoprazole. After this intervention, his serum sodium level declined again to 133 mmol/L. We concluded that both omeprazole and pantoprazole induced syndrome of inappropriate antidiuretic hormone secretion in this patient.

**Conclusion:**

Hyponatremia is worrisome and awareness of medication-induced hyponatremia, especially due to proton pump inhibitors, is needed. In our case, sequential hyponatremia occurred with two different proton pump inhibitors, suggesting a class effect. Therefore, when syndrome of inappropriate antidiuretic hormone secretion due to a proton pump inhibitor is diagnosed, preferably no other medication from the same class is prescribed. When after consideration another proton pump inhibitor is prescribed, serum sodium concentrations should be monitored.

## Background

Hyponatremia is the most common diagnosed electrolyte disorder [1]. In many cases, it is the result of a relative excess of water in relation to sodium and can be caused or aggravated by many factors, such as underlying diseases, physiological changes, inappropriate fluid therapy, and pharmacotherapy [2]. Hyponatremia is mostly found in hospitalized and older patients, patients with congestive heart failure, patients with cirrhosis or pneumonia, or during the postoperative period [3]. Both acute and chronic hyponatremia are associated with increased mortality [4]. Even mild hyponatremia (130–135 mmol/L) can cause an increased risk of falling, cognitive impairment, and gait disturbance [5]. Severe symptoms of hyponatremia are seizures and coma.

Hyponatremia can often be attributed to medication use [6]. In particular, diuretics (thiazide and thiazide-like drugs) and nervous system drugs (antidepressants, antipsychotic agents, and antiepileptic agents) are known to cause hyponatremia [6].

Proton pump inhibitor (PPI)-induced hyponatremia is not reported frequently [6]. However, because PPIs are prescribed worldwide on a large scale, even rare side effects may have implications for clinical practice. A recent Swedish register-based case–control study did find an association between any newly prescribed PPI and hospitalization due to hyponatremia [7]. The underlying pathophysiological mechanism of PPI-induced hyponatremia is not clearly understood [8]. It is probably due to the syndrome of inappropriate antidiuretic hormone secretion (SIADH). Salt-losing nephropathy caused by acute interstitial nephritis may play a role as well.

To the best of our knowledge, no reports about sequential occurrence of hyponatremia with different PPIs have been published. We describe a case of omeprazole-induced hyponatremia with re-occurrence of hyponatremia with pantoprazole.

## Case presentation

A 67-year-old Caucasian male was referred by his general practitioner to the internal medicine out-patient clinic because of hyponatremia (127 mmol/L) found at routine laboratory examination. He had consulted his general practitioner because of abdominal pains. His medical history revealed colon polypectomy, an inguinal hernia, skin cancer, and reflux esophagitis. Three years prior to this presentation his serum sodium level was 135 mmol/L. His family history was non-contributory; he lived with his family, had a regular job, and used to engage in physical activities daily. He stopped smoking cigarettes almost 20 years ago (after 25 pack years) and did not consume alcohol or drugs. No other symptoms or signs such as vomiting, nausea, diarrhea, altered mental status, focal neurological deficits, or palpitations were present. Besides omeprazole (40 mg daily) for reflux esophagitis he used no medication. On physical examination, he appeared euvolemic and no disorientation or abdominal tenderness was found. His blood pressure was 152/88 mmHg and his pulse rate was 72 beats per minute.

Serum analysis showed a sodium level of 129 mmol/L and osmolarity of 274 mOsmol/kg. Urinary osmolarity was 570 mOsmol/kg and urinary sodium level was 35 mmol/L. Other relevant laboratory results were in the normal range (see Table 1). An abdominal ultrasound examination and a chest X-ray revealed no pathology.

**Table 1:** Blood and urinary analysis of our patient

Analysis	Our patient	Reference value*
Sodium (mmol/L)	129	135–145
Potassium (mmol/L)	5.1	3.5–4.5
Calcium (mmol/L)	2.44	2.10–2.55
Creatinine (μmol/L)	80	≤ 90
Serum osmolarity (mOsmol/kg)	274	270–290
TSH (mU/L)	1.18	0.5–4.70
FT4 (pmol/L)	15.5	10–23
Cortisol (nmol/L)	360	138–635
NT-proBNP (pg/mL)	92	< 125
Alkaline phosphatase (U/L)	55	< 115
GGT (U/L)	29	< 55
ALT (U/L)	20	< 45
AST (U/L)	33	< 35
Urinary osmolarity (mOsmol/kg)	570	300–900
Urinary sodium (mmol/L)	35	

*ALT* alanine-aminotransferase, *AST* aspartate transaminase, *FT4* free thyroxine 4, *GGT* gamma-glutamyltransferase, *NT-proBNP* N-terminal pro B-type natriuretic peptide, *TSH* thyroid-stimulating hormone

*Reference value used in Diakonessenhuis Hospital, Utrecht, The Netherlands

**Fig. 1:**
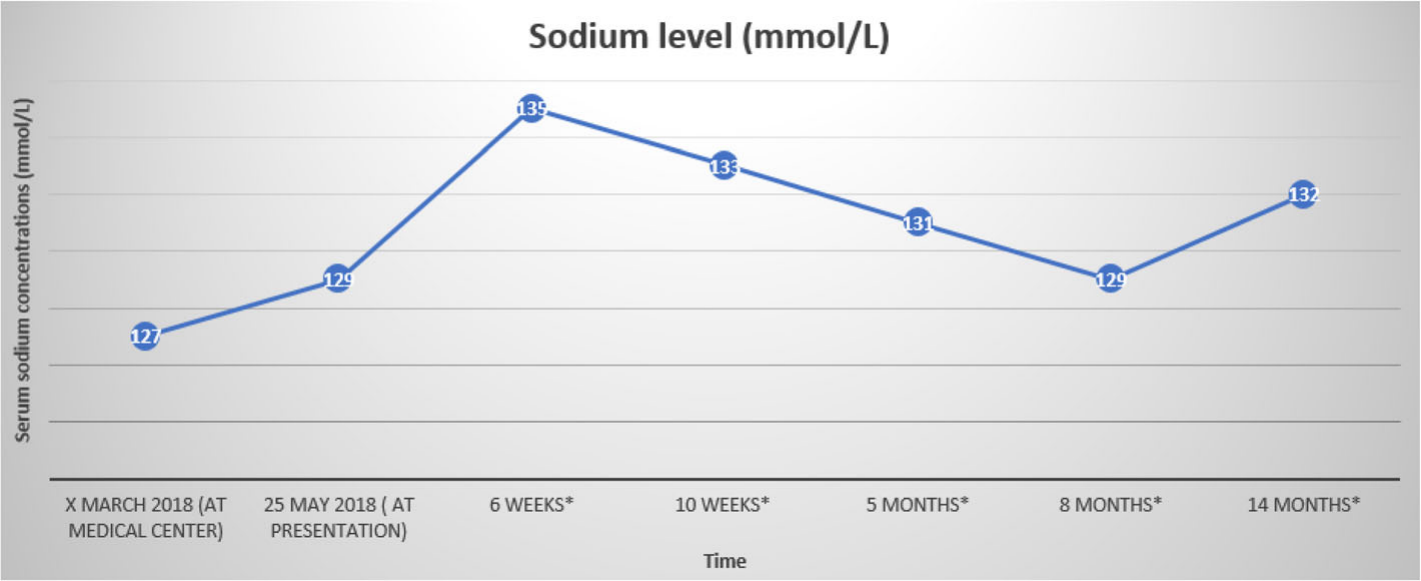
Serum sodium levels at presentation and during follow-up. * after presentation

Hypotonic hyponatremia in an euvolemic patient can be caused by thyroid dysfunction, glucocorticoid deficiency, SIADH, or drugs. Based on the above analysis, we concluded that SIADH due to use of omeprazole caused the hyponatremia. Omeprazole was replaced by an H2 receptor antagonist, ranitidine (150 mg daily). At a follow-up visit after 6 weeks, his sodium level had normalized to 135 mmol/L (see Fig. 1). At this time, he reported persistent reflux complaints, despite ranitidine use. Therefore, ranitidine was discontinued and a different PPI, pantoprazole (40 mg daily) was prescribed. With this therapy, after 4 weeks his serum sodium level had dropped below normal again to 133 mmol/L. We concluded that both omeprazole and pantoprazole caused mild hyponatremia in our patient and considered this to be a PPI class effect.

After careful discussion of risks and benefits with our patient, PPI treatment was continued and after 5 months his serum sodium level declined slightly to 131 mmol/L. During long-term follow-up, his sodium levels were monitored regularly and stayed stable over time (see Fig. 1), without any fluid restriction.

## Discussion

We report PPI-induced hyponatremia occurring with two different agents. Hyponatremia is highly prevalent in both ambulatory and hospitalized patients [1, 5]. A prospective observational study found that hyponatremia in older patients with fragility fractures is mostly multifactorial, whereby dehydration and PPI use were the most associated factors [9]. The authors acknowledged that PPI-induced hyponatremia is infrequently reported and that their results possibly reflect the high prevalence of PPI prescribing in older hospitalized patients [9]. In other studies, PPI-induced hyponatremia has been reported as a rare cause of drug-induced hyponatremia [6, 10]. Unfortunately, we cannot exclude pseudohyponatremia in this case completely. Although highly unlikely, glucose, triglycerides, and lipids were not measured because our patient’s medical history did not reveal diabetes or hypercholesterolemia and his general practitioner had measured a normal glucose prior to the referral. PPIs are worldwide prescribed drugs and on short-term use PPIs are regarded as effective and safe. However, with long-term use several adverse effects are reported, such as a changed gut microbiome, fundic mucosal hypertrophy, *Clostridium difficile* infection, vitamin B12 deficiency, hypomagnesemia, and acute interstitial nephritis [11]. There are only a few reports on PPI-induced hyponatremia with either omeprazole or pantoprazole [12–15]. Durst *et al.* reported the re-occurrence of hyponatremia after reintroducing omeprazole [12]. To the best of our knowledge, this is the first report of hyponatremia occurring after the use of a second PPI. This is of clinical relevance because not only are PPIs one of the most prescribed drugs, but also, when PPI-induced hyponatremia is diagnosed, the clinician should be aware that switching to another PPI potentially does not solve the problem.

## Conclusion

In conclusion, whenever hyponatremia induced by a PPI is diagnosed, the clinician should be aware that switching to another PPI could also lead to hyponatremia. The indication for PPI should be carefully weighed, and alternative therapy should be considered. If PPI therapy is continued, serum sodium levels should be checked regularly.

## Data Availability

Not applicable.

## Abbreviations

SIADH: Syndrome of inappropriate antidiuretic hormone secretion
PPI: Proton pump inhibitor
